# Disease stage predicts post-diagnosis anxiety and depression only in some types of cancer

**DOI:** 10.1038/bjc.2011.503

**Published:** 2011-11-17

**Authors:** A Vodermaier, W Linden, R MacKenzie, D Greig, C Marshall

**Affiliations:** 1 Department of Psychology, University of British Columbia, 2136 West Mall, Vancouver, British Columbia V6T 1Z4, Canada; 2 Department of Obstetrics and Gynecology, Campus Grosshadern, University of Munich, Munich, Germany; 3 BC Cancer Agency, Vancouver, Canada

**Keywords:** anxiety, depression, disease stage, cancer type, gender, age

## Abstract

**Background:**

We hypothesised that patients with advanced disease or a cancer type that has a poor prognosis may be more likely to report anxiety and depressive symptoms after diagnosis; younger age and female gender may moderate these effects.

**Methods:**

Patients (*n*=3850) were consecutively assessed with PSSCAN, a standardised, validated tool, at two large cancer centres between 2004 and 2009.

**Results:**

Female patients reported more anxiety and depressive symptoms (*P*=0.003 to *P*<0.001) compared with men and a healthy comparison group. Older age was associated with fewer anxiety (*P*=0.033 to *P*<0.001) and fewer depressive symptoms (*P*<0.001), but this was not true for lung cancer. Presence of metastases was associated with more anxiety symptoms in patients with gastrointestinal (*P*=0.044; *R*^2^Δ=0.001), lung (*P*=0.011; *R*^2^Δ=0.016), and prostate (*P*=0.032; *R*^2^Δ=0.008) cancer, but this was not true for breast cancer. Furthermore, early disease stage was associated with fewer depressive symptoms among older prostate cancer patients (*P*=0.021; *R*^2^Δ=0.008). Men with early lung cancer reported fewer anxiety (*P*=0.020; *R*^2^Δ=0.013) and depressive (*P*=0.017; *R*^2^Δ=0.016) symptoms than men with advanced disease or women.

**Conclusion:**

As hypothesised, disease stage was directly associated with emotional distress, except for patients with breast cancer. Furthermore, age and gender moderated some of these effects.

A recent meta-analysis (Singer *et al*, 2009) has shown that approximately one-third of patients with cancer suffer from a mental disorder at various stages in the disease trajectory. Given that patients are remarkably heterogeneous in how they respond emotionally to cancer diagnosis and treatment, person and disease characteristics need to be identified that can best explain differential levels of emotional distress. Several small- and medium-scale studies on this topic exist and have for example revealed particularly high levels of depression in patients with lung cancer (Hopwood and Stephens, 2000; Néron *et al*, 2007; Castelli *et al*, 2009), but comparison studies with sufficient statistical power to examine various potential predictor and moderator variables are scarce.

Consequently, we examined relationships of disease stage, age, gender, and their interactions on anxiety and depressive symptoms after diagnosis in a large cohort of cancer patients of four common types of cancers, namely gastrointestinal (GI), lung, breast, and prostate cancer. These distinct cancer types vary in terms of their prognosis as well as the specific implications and subjectively perceived threats of the disease. Based on the current *status quo* of the literature, we hypothesised that: (1) female cancer patients will report more anxiety and depressive symptoms than men, (2) both anxiety and depressive symptoms will be higher when the disease is metastasised and the disease has a poor prognosis such as lung cancer, and (3) age and gender moderate the relationship between disease stage and anxiety and depressive symptoms such that younger age and female gender are associated with higher levels of anxiety and depressive symptoms in advanced disease.

## Materials and methods

### Procedures

The data were collected via a routine screening programme and represent a subsample of a previous, large sample study reporting levels of anxiety and depression of a mixed patient cohort (Vodermaier *et al*, 2010).

For 5139 of the original sample of 10 153 patients (50.6% Vodermaier *et al*, 2010), reliable information on metastasis stage (M0 *vs* M1) was available, and 4156 of these patients belonged to one of the four cancer types under study. Differences in demographic and psychological variables between the total sample and the subsample under study were very minor. Incomplete clinical data may be affected by varying degrees of physician ‘buy-in’ to the electronic record system. Completion rates for psychological assessment are estimated to be ∼90% based on previous studies. Only the data on breast, GI, lung, and prostate cancer patients were of interest in the present study to assure adequate statistical power for the conduct of interaction tests among hypothesised determinants of distress.

### Measure

The 21-item Psychosocial Screen for Cancer (PSSCAN) (Linden *et al*, 2005, 2009) assesses anxiety and depressive symptoms and was specifically developed for use with cancer patients. Details on the questionnaire's reliability and validity including empirically derived cutoffs and norms have been reported (Linden *et al*, 2005, 2009).

### Statistical analysis

One-sample and independent-sample *t*-tests, ANOVAs, and *χ*^2^ tests were conducted to test differences and associations between groups and for comparisons with normative data. For prediction of anxiety and depressive symptoms, hierarchical linear regression was chosen because of unequal and empty cell distributions. First, demographic predictors (age, gender (if applicable)), then early *vs* advanced disease (M0 *vs* M1 also referred to as ‘disease stage’), and finally the interaction terms of disease stage by age and disease stage by gender (if applicable) were entered. Simple slope tests were conducted to test for the significance of eventual interaction effects. All statistical tests were two sided and considered significant at *P*<0.05.

## Results

### Sample

Patients were 60.7 years of age on average; 65% of the sample was female. The proportion of patients with advanced disease was 12.7% across all cancer types (49.0% of lung, 24.9% for GI, 2.6% for breast, and 6.3% for prostate cancer).

### Anxiety and disease stage

**Figure 1:**
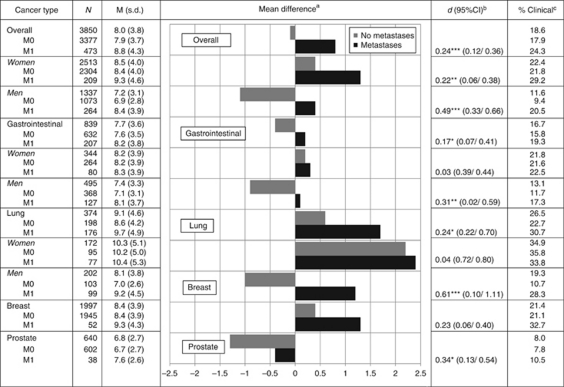
Means and prevalence rates of clinical anxiety by tumour type, stage, and gender. ^a^The difference from the grand mean. ^b^The difference between patients diagnosed primary metastasised *vs* not within each subcategory. ^c^Percentage of patients scoring above the clinical threshold of 11. ^*^P<0.05, ^**^P<0.01, ^***^P<0.001 on *t*-test.

Mean levels of anxiety were in the subclinical range. Women reported more anxiety than men (22.4% *vs* 11.6% Figure 1).

In patients with GI cancers, younger age (*β*=−0.042, *P*=0.001), female gender (*β*=−0.829, *P*<0.001), and advanced disease stage (*β*=0.568, *P*=0.044) were associated with more symptoms of anxiety (*R*^2^=0.045; Table 1).

Lung cancer patients were more anxious compared with the total sample and represented the most anxious group among the four types of cancers investigated. Younger age (*β*=−0.045, *P*=0.033), female gender (*β*=−2.150, *P*<0.001), and advanced disease stage (*β*=1.174, *P*=0.011) were associated with more symptoms. Men with early disease were the least anxious (*β*=2.134, *P*=0.020, *R*^2^=0.098). Although presence of metastasis predicted levels of anxiety in men (*β*=2.189, *P*<0.001), this relationship was not significant for women with lung cancer (*β*=0.171, *P*=0.829).

Mean levels of anxiety in breast cancer patients were higher than in the total sample (t(1680)=4.50, *P*<0.001) and higher than for prostate and GI, but lower than lung cancer. Younger age (*β*=−0.063, *P*<0.001) predicted higher levels of anxiety (*R*^2^=0.044), but neither disease stage (metastasis yes/no) nor its interaction with age were significant.

Men with prostate cancer showed the lowest mean levels of anxiety compared with the total sample (t(743)=−12.8, *P*<0.001) and all other cancer types. Younger age (*β*=−0.055, *P*<0.001) and advanced disease stage (*β*=−1.029, *P*=0.021) were independently associated with higher levels of anxiety (*R*^2^=0.036).

### Depression and disease stage

**Figure 2:**
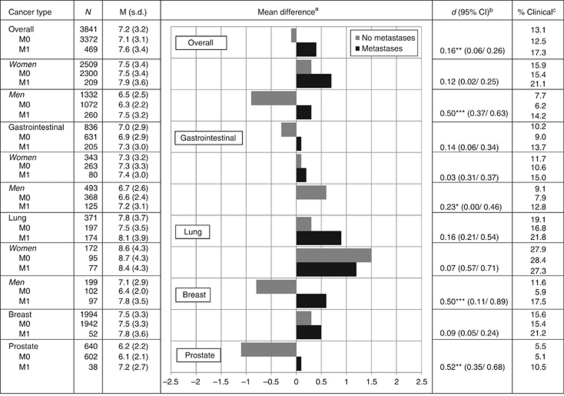
Means and prevalence rates of clinical depression by tumour type, stage, and gender. ^a^The difference from the grand mean. ^b^The difference between patients diagnosed primary metastasised *vs* not within each subcategory. ^c^Percentage of patients scoring above the clinical threshold of 11. ^*^P<0.05, ^**^P<0.01, ^***^P<0.001 on *t*-test.

Mean depression levels of the total sample were slightly below the subclinical threshold (Figure 2). Women were more depressed than men (15.9 *vs* 7.7%).

Patients with GI cancers showed comparable rates of depression to that of the total sample, less than breast and lung cancer patients, but more than prostate cancer patients (F(3, 3564)=365.4, *P*<0.001). Younger age (*β*=−0.030, *P*<0.001) and female gender (*β*=−0.542, *P*=0.007) were associated with higher levels of depression (*R*^2^=0.033).

Lung cancer patients were more depressed than other types of cancer except for breast cancer. Female patients were more depressed (*β*=−1.507, *P*<0.001). Gender also moderated effects of disease stage on depression (*β*=1.791, *P*=0.017) such that men with lung cancer who had early disease experienced the lowest levels of depression (*R*^2^=0.072). Disease stage predicted depression in men (*β*=1.465, *P*<0.001) but not in women with lung cancer (*β*=−0.288, *P*=0.660).

In breast cancer patients, younger age predicted depressive symptoms (*β*=−0.043, *P*<0.001), but disease stage was unrelated (*R*^2^=0.026).

Men with prostate cancer showed the lowest mean depression levels. Depression was related to younger age (*β*=−0.055, *P*<0.001) and presence of metastases (*β*=1.007, *P*=0.006). Older age predicted less depressive symptoms in early (*β*=−0.055, *P*<0.001) but not in advanced disease (*β*=0.027, *P*=0.529) (*R*^2^=0.056).

## Discussion

Cancer site, cancer stage (defined as presence/absence of metastases), age, and gender were associated with different levels of distress but there was no simple, uniform pattern of results. Consistent with predictions, distress was highest in lung cancer patients, which is largely attributable to the high prevalence of metastasised tumours and the poor prognosis.

Beyond that, men with early stage lung cancer experienced fewer anxiety and depressive symptoms compared with men with advanced disease or women independent of their disease stage. This interaction effect, although small in nature, has been identified here for the first time. Surprisingly, presence of metastases was not associated with anxiety and depression in women with breast or GI cancer. This result is inconsistent with a previous study (Simon *et al*, 2009) where it had been shown that in one GI subtype, colon cancer, female patients were more depressed when they had advanced disease. Overall, our findings indicate that cancer type may exert a strong influence on the relationship between disease stage and anxiety or depression.

Although the role of gender as a moderator could only be directly studied in two cancer types, results of sex-specific cancers as breast and prostate cancer also support the notion that men and women adjust differently to the provision of prognostic information.

Normative data for PSSCAN obtained from a healthy cohort (Linden *et al*, 2009) revealed no difference when contrasted with our cancer samples relative to the effects of gender or age on anxiety, and gender on depression. This observation supports the interpretation that the effects seen here are indeed specific to certain types of cancer. Thus older patients report less distress following a diagnosis of cancer. The fact that age was unrelated to depression in lung cancer may be attributable to the particularly poor prognosis. In contrast, in breast cancer patients, younger age is a well-known risk factor for emotional distress (Ell *et al*, 2005; Mehnert and Koch, 2008), and is associated with other factors arising as a consequence of treatment such as threats to body image, sexuality, and fertility, which are interrelated with the age of patients.

The explained variance ranged from 3 to 10% and was highest for prediction of anxiety in lung cancer patients. Apart from cancer site-specific effects, a number of other intraindividual factors not measured here may affect a differential patient stress response. Among these factors are premorbid psychological functioning, cognitions about the meaning of the disease, threat to length of life, threat of anticipated side effects of treatment, or anticipated suffering.

Whether the observed differential relationships of disease stage and the psychological adjustment in certain types of cancer are stable across the entire cancer trajectory awaits further empirical investigation.

### Ethical approval

The study was approved by the Institutional Review Board of the British Columbia Cancer Agency.

## Figures and Tables

**Table 1:** Results of hierarchical linear regression modelling

			**Anxiety**				**Depression**			
**Cancer type**		**Variables**	***β***	***P***	***R*^2^Δ**	***R* ^2^**	***β***	***P***	***R*^2^Δ**	***R* ^2^**
Gastrointestinal	1	Age	−0.043	<0.001	0.038	0.038	−0.030	<0.001	0.028	0.028
		Gender	−0.814	0.001			−0.533	0.008		
	2	Age	−0.042	<0.001	0.005	0.043	−0.030	<0.001	0.003	0.031
		Gender	−0.829	0.001			−0.542	0.007		
		Disease stage	0.568	0.044			3.82	0.097		
	3	Age	−0.030	0.401	0.003	0.045	−0.038	0.201	0.002	0.033
		Gender	−10.028	<0.001			−0.695	0.003		
		Disease stage	0.070	0.876			−0.002	0.997		
		Disease stage × age	−0.007	0.734			0.005	0.781		
		Disease stage × gender	0.828	0.150			0.632	0.179		
										
Lung	1	Age	−0.050	0.018	0.068	0.068	−0.030	0.079	0.050	0.050
		Gender	−20.097	<0.001			−1.481	<0.001		
	2	Age	−0.045	0.033	0.016	0.084	−0.028	0.107	0.007	0.056
		Gender	−2.150	<0.001			−1.507	<0.001		
		Disease stage	1.174	0.011			0.603	0.109		
	3	Age	−0.066	0.342	0.013	0.098	−0.002	0.969	0.016	0.072
		Gender	−3.138	<0.001			−2.349	<0.001		
		Disease stage	0.008	0.990			−0.362	0.510		
		Disease stage × age	0.012	0.777			−0.018	0.614		
		Disease stage × gender	2.134	0.020			1.791	0.017		
										
Breast	1	Age	−0.064	<0.001	0.041	0.041	−0.043	<0.001	0.025	0.025
	2	Age	−0.064	<0.001	0.002	0.043	−0.043	<0.001	0.000	0.026
		Disease stage	0.999	0.064			0.462	0.318		
	3	Age	−0.063	<0.001	0.001	0.044	−0.043	<0.001	0.000	0.026
		Disease stage	1.155	0.035			0.515	0.273		
		Disease stage × age	−0.083	0.084			−0.027	0.508		
										
Prostate	1	Age	−0.053	<0.001	0.028	0.028	−0.046	<0.001	0.032	0.032
	2	Age	−0.055	<0.001	0.008	0.036	−0.048	<0.001	0.016	0.048
		Disease stage	10.029	0.021			1.169	0.001		
	3	Age	−0.057	<0.001	0.001	0.036	−0.055	<0.001	0.008	0.056
		Disease stage	0.976	0.032			10.007	0.006		
		Disease stage × age	0.027	0.541			0.083	0.019		
